# Association between the Intake/Type of Cheese and Cognitive Function in Community-Dwelling Older Women in Japan: A Cross-Sectional Cohort Study

**DOI:** 10.3390/nu16162800

**Published:** 2024-08-22

**Authors:** Takao Suzuki, Yosuke Osuka, Narumi Kojima, Hiroyuki Sasai, Kentaro Nakamura, Chisato Oba, Mayuki Sasaki, Hunkyung Kim

**Affiliations:** 1 National Center for Geriatrics and Gerontology, 7-430 Morioka, Obu 474-8511, Aichi, Japan; suzutaka@ncgg.go.jp; 2 Department of Frailty Research, Center for Gerontology and Social Science, Research Institute, National Center for Geriatrics and Gerontology, 7-430 Morioka, Obu 474-8511, Aichi, Japan; osuka@ncgg.go.jp; 3 Research Team for Promoting Independence and Mental Health, Tokyo Metropolitan Institute for Geriatrics and Gerontology, 35-2 Sakae-cho, Itabashi 173-0015, Tokyo, Japan; nkojima@tmig.or.jp (N.K.); sasai@tmig.or.jp (H.S.); 4 Health Science Research Unit, R&D Division, Meiji Co., Ltd., 1-29-1 Nanakuni, Hachioji 192-0919, Tokyo, Japan; kentarou.nakamura@meiji.com (K.N.); chisato.ohba@meiji.com (C.O.); mayuki.sasaki@meiji.com (M.S.); 5 Gaon Research Center, 402 Pangyo Medical Tower, 142, Unjung-ro, Bundang-gu, Seongnam-shi 13466, Gyeonggi-do, Republic of Korea

**Keywords:** cheese intake, Camembert cheese, MMSE score, mild cognitive decline, community-dwelling older women

## Abstract

While many studies have described the association between cognitive decline and eating habits, little attention has been paid to its association with cheese intake. In this epidemiological study of 1035 community-dwelling women aged ≥ 65, we investigated the association between intake/type of cheese and cognitive function. The anthropometry, functional ability, and the frequency of food intake, including cheese, were assessed. The mini-mental state examination (MMSE) was used to assess cognitive function, and a score of 20–26 was operationally defined as mild cognitive decline. We found that the MMSE score was significantly different between the presence of cheese intake and not (cheese intake: 28.4 ± 1.9; non-cheese intake: 27.6 ± 2.4) and between those who consumed Camembert cheese and those who did not (Camembert cheese: 28.7 ± 1.4; others: 28.3 ± 2.0). After adjusting for confounders, multiple logistic regression identified four independent variables significantly associated with mild cognitive decline: Camembert cheese intake (odds ratio = 0.448, 95% confidence interval = 0.214–0.936), age, usual walking speed, and repetitive saliva swallowing test scores. Our results, while based on cross-sectional data from Japanese community-dwelling older women, identified the significant inverse association between Camembert cheese intake and mild cognitive decline.

## 1. Introduction

As the global population ages, it is crucial to extend a healthy longevity and support older adults in leading independent lives. Maintaining both physical and cognitive functions at high levels is essential to achieve this goal. To sustain cognitive function in older age, staying socially active, continuing to learn, exercising regularly, and eating a healthy diet are key strategies of lifestyle choices and practices [1,2,3]. High-quality cross-sectional, longitudinal, and interventional studies have investigated the impact of diet and nutrition on cognitive function. For instance, many studies have suggested that a dietary pattern rich in milk and dairy products can help prevent dementia and cognitive decline [4,5,6,7]. However, some studies have found no association, resulting in a lack of consensus. The recent systematic reviews on the effect of milk and other dairy products on the risk of cognitive performance decline in older individuals reported that such associations cannot be firmly established owing to remarkable heterogeneity in the methodology used among the observational studies [8,9]. 

Many previous studies have highlighted the effect of consuming dairy products on suppressing cognitive decline and dementia onset [7,10]. However, many types of dairy products are available (such as those with high or low fat, fermented or not). Thus, describing the association between the cognitive function of older individuals and dairy as a whole is challenging. For example, some studies have reported that a high intake of full-fat dairy or saturated-fat dairy increases the risk of MCI, Alzheimer’s disease, dementia, psychomotor retardation, and global cognitive dysfunction, whereas low-fat dairy consumption was shown to have beneficial effects [11].

Of the numerous studies on different dairy products, some studies have focused on the association of cheese consumption and two brain substances, namely β-amyloid and brain-derived neurotrophic factor (BDNF), that play important roles in cognitive function. For example, a randomized controlled trial on community-dwelling older adults with mild cognitive impairment (MCI) has reported that Camembert cheese intake increases the BDNF levels [12]. Moreover, previous studies have demonstrated that decreasing the β-amyloid and increasing the BDNF levels are effective and essential measures for preventing cognitive decline and Alzheimer’s disease (dementia) [10,12,13,14]. 

Based on these findings, this study aimed to explore the association between cheese intake/type and cognitive function, assessed using the mini-mental state examination (MMSE) in a Japanese observational cohort of community-dwelling older women. 

## 2. Materials and Methods

### 2.1. Study Participants

The participants in this study were 1035 community-dwelling women aged ≥ 65 years who participated in a cohort of “the Otassha study”, a comprehensive health examination conducted by the Tokyo Metropolitan Institute for Geriatrics and Gerontology in 2017. The participant selection process has been described in detail in our previous study [15] and is briefly described here. To ensure community representativeness, 6788 older women aged ≥ 65 years (approximately 10% of the total female population of Itabashi) were randomly selected using the Basic Resident Register in 2017. Subsequent to the exclusion of 422 women participating in other cohort studies, invitation letters were sent to 6366 women. A total of 1035 women participated in the health examination in 2017. 

This study was conducted under the approval of the Clinical Research Ethics Committee of the Tokyo Metropolitan Institute for Geriatrics and Gerontology (ID R2-25, approval date: 24 May 2017). All participants signed an informed consent after the procedure of this research was described completely. 

### 2.2. Measures

#### 2.2.1. Measurement of Anthropometric and Physical Function

Height and body weight were measured and used to derive the body mass index. The calf circumference and grip strength (using a hand-held Smedley-type dynamometer, Takei Scientific Instruments Co., Ltd., Niigata, Japan) were measured [10], with the greater strength of two trials recorded. Usual walking speed was measured on a flat walking path of 11 m with indicators placed at the 3 m and 8 m mark. A stopwatch was used to measure the time taken to walk the 5 m distance between the indicators, and the faster time of two trials was recorded. Assistive walking devices were allowed when the participant expressed concerns about walking without a device or when the investigators suspected a risk of falling. Swallowing function was assessed using the repetitive saliva swallowing test (RSST) by placing the fingers around the laryngeal protuberance to calculate the number of times saliva has been swallowed within 30 s.

#### 2.2.2. Interview Survey

Interviews were conducted face to face to assess geriatric depression scale (GDS) scores, frequencies of food intake, history of falls, urinary incontinence, and chronic diseases and conditions, such as heart disease, hyperlipidemia, dyslipidemia, diabetes, osteoporosis, osteoarthritis, and anemia. 

In order to investigate the frequency of food intake and to calculate the dietary variety score (DVS), participants completed a food-group-based dietary questionnaire called food frequency questionnaire (FFQ) [15]. FFQ is a questionnaire developed in Japan with simple questions on the respondents’ daily food intake based on 10 categories: meats, fish and shellfish, eggs and egg products, soybeans and soybean products, milk, seaweeds, vegetables, fruits, potatoes, and oils. 

DVS scores were calculated as the total score of how frequently an individual consumed food, by assigning 1 point for a response of “eat almost every day” and 0 for “eat once every two days/eat once or twice a week/eat hardly ever” for each of the 10 food item groups and showing the sum. This investigation had been previously performed by Kumagai et al. in 2003 [15]. After the relationship between the DVS scores and higher life functions had been reported, the significance of this questionnaire has been tested by a number of researchers [16,17]. Later, the questionnaire has also been validated in the Japanese population by Nakamoto et al. [18]. In this research, the DVS was calculated from foods consumed across the 9 categories. Out of the 10 original categories, milk was excluded since milk and cheese were both treated as the same family, which is dairy, in the present study. The total score ranged from 0 to 9 points, calculated in the same way as the original DVS scores.

Cheese intake frequency and type were also investigated. The response regarding which cheese frequency was determined in the same way as the FFQ, selecting one of “eat almost every day”, “eat once every two days”, “eat once or twice a week”, or “eat hardly ever”. The type of cheese consumed was selected from one or more of the types listed: processed cheese, fresh cheese, Camembert cheese, blue cheese, or other cheese. According to the results of the survey, participants consuming cheese at least once or twice a week were classified into the “cheese intake” group, whereas others were classified into the “non-cheese intake” group. Also, considering the type of cheese consumed, the participants were also categorized into the “Camembert cheese” and “other cheese” groups, which included participants who were categorized into the cheese intake group but did not consume Camembert cheese.

#### 2.2.3. Blood Indicators

Non-fasting blood samples were taken and analyzed centrally in one laboratory (SRL, Inc., Tokyo, Japan). Serum creatinine and lipid levels (total cholesterol, high-density lipoprotein [HDL] cholesterol, and triglycerides) were determined by an enzymatic assay. Serum albumin and glycated hemoglobin (HbA1c) were measured by the bromocresol green method and latex agglutination assay, respectively.

#### 2.2.4. Cognitive Function

The MMSE was used to assess the global cognitive status. Although many measurements are employed for cognitive function, MMSE is the most widely used cognitive measurement tool. Mild cognitive decline was operationally defined as MMSE scores of 20–26 [19,20]. Meanwhile, participants with an MMSE score ≤ 19 were excluded from the analysis due to suspected middle-stage to moderate dementia.

### 2.3. Data Analysis

Descriptive statistics are expressed as the mean and standard deviation or frequency (%). Participants were classified into two groups based on the responses to the interview survey or MMSE scores. Participants who responded “daily”, “once every two days”, or “once or twice a week” were classified into the cheese intake group and those who responded “no intake” were classified into the non-cheese intake group. In the cheese intake group, those who answered that they consumed Camembert cheese were allocated into the Camembert cheese intake group, and the others who answered yes to consuming other cheese but not Camembert cheese were grouped into the other cheese intake group. Classifications based on the MMSE scores were as follows: those with the scores of ≥27 and those in the range of 20 and 26. The Student’s *t*-test was used for continuous variables, and the chi-square test was used for categorical variables. 

Multiple logistic regression analyses were used to analyze factors associated with mild cognitive decline. The dependent variable was an MMSE score 26 and under and 20 and above (20 ≤ MMSE score ≤ 26). Model I included only the cheese type and intake status. Model II was adjusted for age, physical function, and physique factors. Model III was further adjusted for medical history, blood variables, swallowing function, urinary incontinence, depressive symptoms, and milk intake. Each of the variables listed above were entered into the multiple logistic regression models to obtain the odds ratio (OR) and 95% confidence interval (CI). 

*p*-Values less than 0.05 were considered statistically significant. All analyses were performed using the Statistical Package for Social Sciences (SPSS) version 25.0 (SPSS Inc., Tokyo, Japan).

## 3. Results

A total of 1035 women participated in this study. Of the 1035 women, 883 (85.3%) and 150 (14.5%) were categorized in the “cheese intake” and “non-cheese intake” groups, respectively (Table 1). Among the 883 women categorized as the cheese intake group, 30.7%, 27.4%, and 36.6% responded that their frequency of cheese intake was “daily”, “once every 2 days”, and “once or twice a week”, respectively. Data on the frequency of cheese intake on 5.3% of the participants who answered yes to regular cheese intake were missing. 

In contrast to the cheese intake group, the non-cheese intake group had a smaller calf circumference, smaller grip strengths, slower usual walking speed, lower DVS, higher GDS scores, a lower percentage of milk consumers, and lower total MMSE scores, including the MMSE sub-scale scores for temporal orientation, registration, attention and calculation, and remote memory (Table 2). 

**Figure 1:**
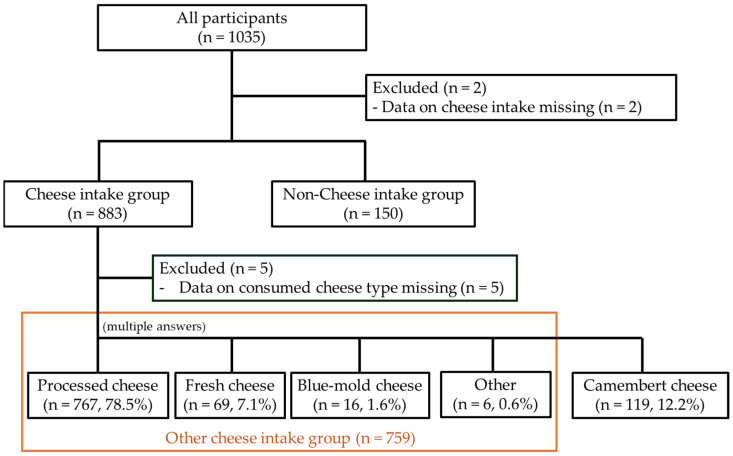
A flow-chart of the participant enrollment in this observational study. Participants were classified into each group according to their response to the survey.

The type of cheese consumed was investigated in the 883 participants who answered yes to having regular cheese intake through a multiple-answer question. A total of 977 valid responses were obtained. The majority (78.5%) consumed processed cheese, whereas fresh cheese, blue mold cheese, and Camembert cheese were consumed by 7.1%, 1.6%, and 12.2%, respectively (Figure 1). According to these data, 119 participants who answered yes to having regular Camembert cheese intake were classified into the Camembert cheese intake group, and the other 759 participants who answered yes to consuming cheese other than Camembert cheese were classified into the other cheese intake group (Figure 1). Data on the consumed cheese type of the remaining five participants were missing.

To characterize the groups categorized according to the type of cheese consumed, the factors associated with Camembert cheese intake were analyzed. Table 3 shows a comparison between the Camembert cheese intake and other cheese intake groups. The Camembert cheese intake group had a smaller calf circumference, a higher total MMSE score, and higher scores for the MMSE sub-scales of temporal orientation, attention and calculation, and other functions.

**Figure 2:**
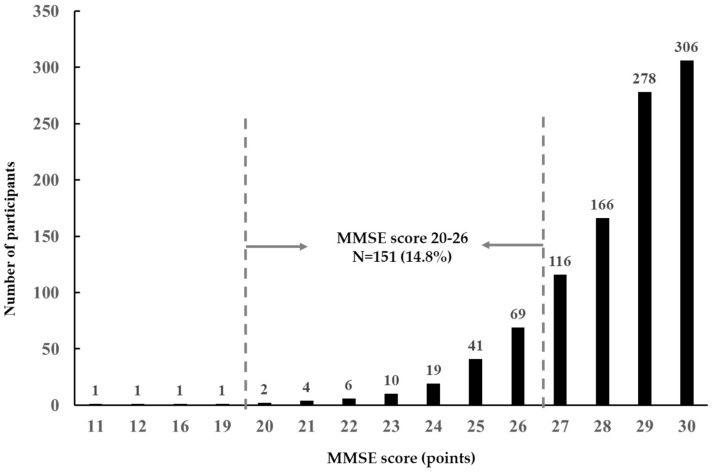
Distribution of mini-mental state examination scores. N = 1021.

Figure 2 shows the distribution of MMSE scores among the study participants. A total of 866 (84.8%) participants had an MMSE score ≥ 27, and 151 (14.8%) had an MMSE score of 20–26. Four (0.4%) women with an MMSE score ≤ 19 were excluded from the analysis.

We investigated the factors related to the MMSE scores by comparing the group with MMSE scores ≥ 27 and those with MMSE scores of 20–26. Table 4 shows a comparison of the measured variables between the group with MMSE scores ≥ 27 and those with MMSE scores of 20–26. Compared to the group with MMSE scores ≥ 27, the group with MMSE scores of 20–26 was older, with a smaller calf circumference, weak grip strength, slower usual walking speed, lower RSST scores, lower albumin levels, and higher GDS scores. 

Finally, we aimed to reveal the factors that are correlated with the cognitive ability by adjusting the dataset under different conditions. As is shown in Table 5, multiple logistic regression (Model III) identified four significant independent variables for mild cognitive decline: Camembert cheese intake (OR = 0.448, 95% CI = 0.214–0.936), age (OR = 1.114, 95% CI = 1.059–1.171), usual walking speed (OR = 0.260, 95% CI = 0.109–0.621), and RSST scores (OR = 0.865, 95% CI = 0.750–0.995). In all three models, Camembert cheese intake was significantly associated with mild cognitive decline.

## 4. Discussion

In this study, we analyzed the association between the type and frequency of cheese consumption and cognitive function using cross-sectional data from randomly selected community-dwelling older women. Our results suggest that Camembert cheese intake may prevent mild cognitive decline (MMSE scores of 20–26) (OR = 0.448). Here, we will examine the significant results shown in this study from multiple aspects.

Of the numerous studies on different dairy products, many have explored the association between cheese intake and cognitive function. Regarding cheese consumption, Rahman et al. found that cheese intake was inversely associated with cognitive impairment in a simple logistic regression analysis by them (OR = 0.59, 95% CI = 0.42–0.84, *p* = 0.003) [21]. Findings from a UK Biobank study conducted by Klinedinst et al. in 2020 demonstrated that daily cheese intake strongly predicted better fluid intelligence test scores over time (FH: β = 0.207, *p* < 0.001) [22]. A Canadian longitudinal study performed by Tessier et al. revealed that cheese intake was positively associated with the executive function domain and verbal fluency [23]. Zhang et al. showed in a meta-analysis that dementia was one of the several health outcomes related to cheese consumption [24]. In an Italian case–control study by Filippini et al., fresh cheese intake was found to be positively associated with early-onset frontotemporal dementia (EO-FTD) but not with early-onset Alzheimer’s dementia (EO-AD) [25]. Conversely, in the same study, aged cheese intake did not show any association with EO-FTD but had a slight positive association with EO-AD. Other studies have also questioned the association between cheese and cognition. de Goeij et al. [26] reported that cheese intake was associated with information processing speed but not with memory, suggesting that the influence may vary based on the cognitive function sub-scale. Ni et al. suggested that the consumption of dairy products and cognition showed no clear associations [27]. Dobreva focused on the relationship between a Mediterranean diet and cognitive function and found positive relationships; however, cheese was not found to be related [28]. Thus, whether cheese affects human cognitive ability positively or not is still controversial.

Existing evidence suggests the possibility that the area where each research is performed may be an important aspect in the debate on the association between cheese and cognition. Recently, a meta-analysis on the association between the amount of cheese intake and cognitive decline/dementia found that studies conducted in Asian countries showed an association between a high intake of dairy products and the prevention of dementia and cognitive decline. In contrast, studies conducted in Europe showed no such association [29]. The discrepancy may be related to the daily intake of dairy products, which ranges from 29 to 165 g/day in Asian regions and 170 to 711 g/day in Europe [30]. In studies conducted in Asian regions, a high intake of dairy products led to a 43% lower risk of cognitive decline [31], whereas in regions with already high intake, no further health benefit was noted [9]. According to a study by Ozawa et al. on the Japanese diet, their mean daily intake of dairy products was 84.6 g, as assessed using a semi-quantitative food frequency questionnaire [7], which is lower than that observed in European regions. Notably, it is undeniable that the results of our study may be influenced by the underlying factor that the Japanese diet consists of a reduced daily intake of dairy products, although it cannot be clarified from the data of this study. Thus, this fact should be taken into consideration when interpreting the results of this study.

Dairy products are a heterogeneous food group, including fermented and nonfermented foods, and the percentage composition of their nutrients, such as fats and sodium, also varies. Dairy products rich in proteins, minerals, vitamins, and essential amino acids are directly or indirectly associated with cognitive function [32]. Among dairy products, fermented products, in particular, are effective in preventing cardiovascular diseases or diabetes [33,34]; moreover, these act as mediators of the association between the use of dairy products and cognitive decline [35]. In 2021, Tessier et al. analyzed data from 7945 men and women aged ≥ 65 years who participated in the Canadian Longitudinal Study on Aging and observed a positive and independent association between total fermented dairy intake and executive function domains, emphasizing the usefulness of fermented dairy intake [23].

In addition, fermented dairy such as cheese could affect older populations in various ways other than cognitive function. For example, results from a multicenter open trial demonstrated that the consumption of fortified soft plain cheese by older women who are deficient in vitamin D possibly could lower markers of bone resorption and thereby attenuate bone loss attributed to aging [36]. Regarding frailty and cardiovascular risk, both of which are frequently encountered among older persons, cheese, being rich in calcium, protein, and certain fats, might have a complex impact on cardiovascular risk and frailty. However, a recent prospective cohort study by Struiji EA et al., analyzing data from 85,280 women aged ≥ 60 years participating in the Nurses’ Health Study, showed that the habitual consumption of milk or yogurt was not associated with risk of frailty, whereas cheese consumption may be associated with an increased risk, probably because of saturated fats and sodium [37]. Regarding the association between dairy consumption and cardiovascular diseases, traditional concerns have focused on the saturated fat content in dairy products, which can raise LDL cholesterol levels, a risk factor for cardiovascular diseases (CVDs). A recent meta-analysis on 27 studies, which included 8648 cases of CVD, 11,806 cases of coronary heart diseases (CHDs), and 29,300 cases of stroke, revealed an inverse association between total dairy intake and CVD (RR = 0.90, 95% CI: 0.81–0.99) and stroke (RR = 0.88, 95% CI: 0.82–0.95). Meanwhile, no association was observed between total dairy intake and CHD [38]. At present, considering that there is no global consensus on the relationship of dairy products with cardiovascular diseases, additional studies are required to investigate the effect of different factors on the association of dairy intake, particularly cheese, which contains larger amounts of saturated fat and sodium, and CVD.

Cheese is also a fermented dairy product and expected to have health benefits through numerous bioactive compounds generated during ripening [39]. In fact, we previously reported the effect of white mold cheese (Camembert cheese) on BDNF in MCI of community-dwelling older Japanese women in an RCT. A significant interaction was observed in BDNF after a three-month intervention of Camembert cheese intake [12].

Upon the fermentation of the white mold *Penicillium camemberti*, functional lipids such as oleamide and dehydroergosterol are produced. Ano et al. pointed out that these components help suppress brain inflammation [13,40]. Furthermore, a recent study has shown that oleamide contributes to maintaining cognitive function and improving sleep in humans [41]. Through these actions, it can be speculated that the intake of white mold cheese, including Camembert cheese, may help suppress mild cognitive decline. However, the mode of action of Camembert cheese or its content on cognitive function cannot be illustrated from the data provided in this research.

Lastly, the present study has several limitations. First, the association between Camembert cheese intake and mild cognitive decline reported in this paper was obtained from an analysis of cross-sectional data. Whether Camembert cheese intake contributes to reducing the risk of mild cognitive decline cannot be elucidated from the present result and must be further investigated by conducting a longitudinal study. Second, information on the status of Camembert cheese intake was based on self-reporting during the interview and was not objectively quantified. Third, despite there being many proposed cut-off values for mild cognitive decline, the cut-off value used in the present study was determined operationally as an MMSE score of 20–26, according to that proposed by Jan Versijpt et al. and Arevalo-Rodriguez et al. [19,20]. Finally, this study was limited to female participants. The percentage of individuals requiring long-term care due to dementia is reportedly higher among women (19.9%) than among men (14.4%) [28], suggesting that women may be more severely affected by cognitive decline. Thus, we performed the analysis by limiting the participants to women. The influence of sex differences on the association between cheese intake and cognitive function should be examined in the future.

## 5. Conclusions

In this cross-sectional study of Japanese community-dwelling older women, our results suggest that Camembert cheese intake is associated with mild cognitive decline even after adjusting for multiple confounding factors. A large-scale longitudinal analysis should be conducted in the future to elucidate the causal relationship.

## Figures and Tables

**Table 1:** Cheese intake of study participants.

Domain	Category	n	%
Cheese Intake	Intake	883	85.3
	No intake	150	14.5
	Unknown	2	0.2
	Total	1035	100.0

**Table 2:** Comparison of selected variables between the cheese intake and non-cheese intake groups.

Variables	Cheese Intake	n	Mean ± SD or Percent	*t*- or Chi-Square Values	*p*-Value
Age, years	No	150	71.6 ± 4.2	0.608	0.272
	Yes	883	71.9 ± 4.5		
**Calf circumference, cm**	**No**	**150**	**34.0 ± 3.0**	**1.873**	**0.031**
	**Yes**	**883**	**34.5 ± 2.9**		
**Grip strength, kg**	**No**	**149**	**20.9 ± 4.1**	**1.963**	**0.025**
	**Yes**	**876**	**21.6 ± 3.9**		
**Usual walking speed, m/s**	**No**	**149**	**1.3 ± 0.3**	**2.897**	**0.002**
	**Yes**	**881**	**1.4 ± 0.3**		
RSST, times/30 s	No	150	3.7 ± 1.8	0.601	0.274
	Yes	875	3.8 ± 1.7		
**DVS, points**	**No**	**149**	**4.0 ± 1.8**	**4.689**	**<0.001**
	**Yes**	**880**	**4.8 ± 2.0**		
Creatinine, mg/dL	No	149	0.67 ± 0.13	1.550	0.061
	Yes	883	0.71 ± 0.29		
Total cholesterol, mg/dL	No	149	223.0 ± 35.3	1.378	0.084
	Yes	836	227.3 ± 35.5		
HDL cholesterol, mg/dL	No	149	68.9 ± 18.0	0.813	0.208
	Yes	883	70.2 ± 18.5		
Triglycerides, mg/dL	No	149	152.5 ± 84.0	0.377	0.353
	Yes	883	155.5 ± 90.7		
Albumin, g/dL	No	149	4.4 ± 0.3	0.352	0.363
	Yes	836	4.4 ± 0.3		
HbA1c, %	No	149	5.6 ± 0.8	0.097	0.461
	Yes	883	5.6 ± 0.6		
**GDS score, points**	**No**	**150**	**3.1 ± 3.0**	**3.586**	**<0.000**
	**Yes**	**882**	**2.1 ± 2.3**		
**MMSE score, points**	**No**	**147**	**27.6 ± 2.4**	**3.452**	**<0.001**
	**Yes**	**874**	**28.4 ± 1.9**		
**Temporal orientation**	**No**	**147**	**4.8 ± 0.6**	**1.904**	**0.029**
	**Yes**	**877**	**4.9 ± 0.4**		
Spatial orientation	No	148	4.9 ± 0.5	1.291	0.099
	Yes	877	4.9 ± 0.3		
**Registration**	**No**	**148**	**3.0 ± 0.1**	**1.692**	**0.046**
	**Yes**	**877**	**3.0 ± 0.2**		
**Attention and calculation**	**No**	**148**	**3.9 ± 1.3**	**2.898**	**0.002**
	**Yes**	**876**	**4.3 ± 1.0**		
**Remote memory**	**No**	**148**	**2.3 ± 0.9**	**2.966**	**0.002**
	**Yes**	**877**	**2.6 ± 0.7**		
Other functions	No	148	8.7 ± 0.5	1.072	0.142
	Yes	875	8.7 ± 0.6		
Number of chronic diseases, N	No	150	2.1 ± 1.5	1.218	0.112
	Yes	878	2.3 ± 1.7		
Diabetes, yes (%)	No	12/150	8.0	0.257	0.612
	Yes	82/883	9.3		
Hyperlipidemia, yes (%)	No	50/150	33.3	0.000	0.993
	Yes	294/883	33.3		
Falls, yes (%)	No	26/150	17.3	2.020	0.155
	Yes	115/883	13.0		
Urinary incontinence, yes (%)	No	64/150	42.7	0.078	0.780
	Yes	366/883	41.4		
**Milk intake, yes (%)**	**No**	**87/150**	**58.0**	**15.288**	**<0.001**
	**Yes**	**650/883**	**73.6**		

Data are presented as mean ± standard deviation (SD) for continuous variables and percentage for categorical variables. RSST: repetitive saliva swallowing test; DVS: dietary variety score; HDL: high-density lipoprotein; HbA1c: hemoglobin A1c; GDS: geriatric depression scale; MMSE: mini-mental state examination; N: number. Analysis was conducted using Student’s *t*-test for continuous variables and chi-square for categorical variables.

**Table 3:** Comparison of selected variables between the Camembert and other cheese type intake groups.

**Variables**	**Category**	**n**	**Mean ± SD** **or Percent**	***t*- or** **Chi-Square Values**	***p*-Value**
Age, years	Other	759	71.8 ± 4.5	0.487	0.313
	Camembert	119	72.0 ± 4.8		
**Calf circumference, cm**	**Other**	**759**	**34.5 ± 2.8**	**1.680**	**0.047**
	**Camembert**	**119**	**34.1 ± 3.2**		
Grip strength, kg	Other	753	21.5 ± 3.8	1.131	0.129
	Camembert	118	22.0 ± 4.2		
Usual walking speed, m/s	Other	757	1.4 ± 0.3	0.892	0.186
	Camembert	119	1.4 ± 0.2		
RSST, times/30 s	Other	752	3.8 ± 1.7	0.770	0.221
	Camembert	118	3.9 ± 1.8		
DVS, points	Other	757	4.8 ± 1.9	0.148	0.441
	Camembert	118	4.8 ± 2.0		
Creatinine, mg/dL	Other	759	0.7 ± 0.3	0.645	0.260
	Camembert	119	0.7 ± 0.1		
Total cholesterol, mg/dL	Other	759	227.4 ± 35.4	0.150	0.440
	Camembert	119	227.9 ± 35.4		
HDL cholesterol, mg/dL	Other	759	70.0 ± 18.5	1.148	0.126
	Camembert	119	72.1 ± 18.1		
Triglycerides, mg/dL	Other	759	156.3 ± 91.8	0.556	0.289
	Camembert	119	151.3 ± 85.0		
Albumin, g/dL	Other	759	4.4 ± 0.3	1.312	0.095
	Camembert	119	4.4 ± 0.3		
HbA1c, %	Other	759	5.6 ± 0.6	0.507	0.306
	Camembert	119	5.5 ± 0.5		
GDS score, points	Other	758	2.2 ± 2.3	1.240	0.108
	Camembert	119	1.9 ± 2.2		
**MMSE score, points**	**Other**	**750**	**28.3 ± 2.0**	**2.527**	**0.006**
	**Camembert**	**119**	**28.7 ± 1.4**		
**Temporal orientation**	**Other**	**753**	**4.9 ± 0.4**	**2.430**	**0.008**
	**Camembert**	**119**	**4.9 ± 0.2**		
Spatial orientation	Other	753	4.9 ± 0.3	0.675	0.250
	Camembert	119	4.9 ± 0.2		
Registration	Other	753	3.0 ± 0.2	0.826	0.205
	Camembert	119	3.0 ± 0.2		
**Attention and calculation**	**Other**	**752**	**4.2 ± 1.1**	**1.827**	**0.035**
	**Camembert**	**119**	**4.4 ± 0.8**		
Remote memory	Other	753	2.5 ± 0.7	0.530	0.298
	Camembert	119	2.6 ± 0.6		
**Other functions**	**Other**	**751**	**8.7 ± 0.6**	**2.255**	**0.013**
	**Camembert**	**119**	**8.8 ± 0.4**		
Number of chronic diseases, N	Other	755	2.3 ± 1.7	0.611	0.271
	Camembert	118	2.4 ± 1.9		
Diabetes, yes (%)	Other	69/759	9.1	0.059	0.807
	Camembert	10/119	8.4		
Hyperlipidemia, yes (%)	Other	249/759	32.8	1.159	0.282
	Camembert	45/119	37.8		
Falls, yes (%)	Other	100/759	13.2	0.029	0.864
	Camembert	15/119	12.6		
Urinary incontinence, yes (%)	Other	311/759	41.0	0.538	0.463
	Camembert	53/119	44.5		
Milk intake, yes (%)	Other	558/759	73.5	0.237	0.626
	Camembert	90/119	75.6		

Data are presented as mean ± standard deviation (SD) for continuous variables and percentage for categorical variables. RSST: repetitive saliva swallowing test; DVS: dietary variety score; HDL: high-density lipoprotein; HbA1c: hemoglobin A1c; GDS: geriatric depression scale; MMSE: mini-mental state examination; N: number. Analysis was conducted using Student’s *t*-test for continuous variables and chi-square for categorical variables.

**Table 4:** The comparison of selected variables between the following groups: MMSE scores ≥ 27 and MMSE scores of 20–26.

Variables	Category	N	Mean ± SD or Percent	*t*- or Chi-Square Values	*p*-Value
**Age, years**	**MMSE score ≥ 27**	**866**	**71.4 ± 4.4**	**6.403**	**<0.001**
	**MMSE score 20–26**	**151**	**73.9 ± 4.3**		
**Calf circumference, cm**	**MMSE score ≥ 27**	**866**	**34.5 ± 2.9**	**2.185**	**0.015**
	**MMSE score 20–26**	**151**	**33.9 ± 2.6**		
**Grip strength, kg**	**MMSE score ≥ 27**	**860**	**21.7 ± 3.9**	**3.800**	**<0.001**
	**MMSE score 20–26**	**150**	**20.4 ± 0.8**		
**Usual walking speed, m/s**	**MMSE score ≥ 27**	**863**	**1.4 ± 0.2**	**5.042**	**<0.001**
	**MMSE score 20–26**	**151**	**1.2 ± 0.3**		
**RSST, times/30 s**	**MMSE score ≥ 27**	**863**	**3.9 ± 1.8**	**2.941**	**0.002**
	**MMSE score 20–26**	**151**	**3.4 ± 1.4**		
DVS, points	MMSE score ≥ 27	864	4.6 ± 1.9	1.087	0.139
	MMSE score 20–26	149	4.8 ± 2.0		
Creatinine, mg/dL	MMSE score ≥ 27	866	0.7 ± 0.3	0.645	0.260
	MMSE score 20–26	151	0.7 ± 0.2		
Total cholesterol, mg/dL	MMSE score ≥ 27	866	227.0 ± 35.8	0.987	0.162
	MMSE score 20–26	151	223.9 ± 32.5		
HDL cholesterol, mg/dL	MMSE score ≥ 27	866	70.3 ± 18.6	1.116	0.132
	MMSE score 20–26	151	68.4 ± 17.2		
Triglycerides, mg/dL	MMSE score ≥ 27	866	155.4 ± 91.0	0.095	0.462
	MMSE score 20–26	151	154.6 ± 83.9		
**Albumin, g/dL**	**MMSE score ≥ 27**	**866**	**4.4 ± 0.3**	**2.304**	**0.011**
	**MMSE score 20–26**	**151**	**4.3 ± 0.3**		
HbA1c, %	MMSE score ≥ 27	866	5.5 ± 0.6	1.152	0.125
	MMSE score 20–26	151	5.6 ± 0.8		
**GDS score, points**	**MMSE score ≥ 27**	**865**	**2.2 ± 2.3**	**1.890**	**0.030**
	**MMSE score 20–26**	**151**	**2.6 ± 2.7**		
Number of chronic diseases, N	MMSE score ≥ 27	861	2.3 ± 1.7	0.080	0.468
	MMSE score 20–26	151	2.3 ± 1.8		
Diabetes, yes (%)	MMSE score ≥ 27	75/866	8.7	1.645	0.200
	MMSE score 20–26	18/151	11.9		
Hyperlipidemia, yes (%)	MMSE score ≥ 27	299/866	34.5	2.599	0.107
	MMSE score 20–26	42/151	27.8		
Falls, yes (%)	MMSE score ≥ 27	113/866	13.0	0.529	0.467
	MMSE score 20–26	23/151	15.2		
Urinary incontinence, yes (%)	MMSE score ≥ 27	363/866	41.9	0.039	0.844
	MMSE score 20–26	62/151	41.1		
Milk intake, yes (%)	MMSE score ≥ 27	624/866	72.1	0.64	0.424
	MMSE score 20–26	104/151	68.9		

Data are presented as mean ± standard deviation (SD) for continuous variables and percentage for categorical variables. RSST: repetitive saliva swallowing test; DVS: dietary variety score; HDL: high-density lipoprotein; HbA1c: hemoglobin A1c; GDS: geriatric depression scale; MMSE: mini-mental state examination; N: number. Analysis was conducted using Student’s *t*-test for continuous categorical variables and chi-square for categorical variables.

**Table 5:** Odds ratio (OR) and 95% confidence interval (CI) for variables associated with MMSE scores of 20–26.

Independent Variable	Model I			Model II			Model III		
	OR	95% CI	*p*-Value	OR	95% CI	*p*-Value	OR	95% CI	*p*-Value
**Type of cheese, Camembert cheese**	**0.484**	**0.238–0.984**	**0.045**	**0.465**	**0.224–0.966**	**0.040**	**0.448**	**0.214–0.936**	**0.033**
Cheese intake, yes	0.816	0.354–1.881	0.634	0.640	0.269–1.521	0.312	0.605	0.252–1.455	0.262
**Age, 1 year**				**1.114**	**1.061–1.170**	**<0.001**	**1.114**	**1.059–1.171**	**<0.001**
Calf circumference, 1 unit				0.972	0.899–1.051	0.476	0.963	0.890–1.042	0.353
Grip strength, 1 unit				0.981	0.923–1.043	0.540	0.989	0.929–1.052	0.722
**Usual walking speed, 1 unit**				**0.259**	**0.113–0.591**	**0.001**	**0.260**	**0.109–0.621**	**0.002**
Diabetes, yes							1.724	0.899–3.304	0.101
Creatinine, 1 unit							0.964	0.548–1.695	0.898
Total cholesterol, 1 unit							1.001	0.995–1.007	0.738
Albumin, 1 unit							1.105	0.488–2.500	0.811
**RSST, 1 unit**							**0.865**	**0.750–0.995**	**0.046**
Urinary incontinence, yes							1.093	0.644–1.854	0.742
GDS score, 1 unit							0.964	0.879–1.057	0.436
Milk intake, yes							0.954	0.601–1.513	0.841

RSST: repetitive saliva swallowing test; GDS: geriatric depression scale.

## Data Availability

The data presented in this study are not publicly available due to ethical and legal restrictions imposed by the Ethics Committee at the Tokyo Metropolitan Institute of Gerontology but are available from the corresponding author upon reasonable request.
